# Case report: Two siblings with very late onset of holocarboxylase synthase deficiency and a mini-review

**DOI:** 10.3389/fgene.2024.1249480

**Published:** 2024-09-26

**Authors:** Margaux Gaschignard, Louis Domenach, Delphine Lamireau, Claire Guibet, Sandrine Roche, Emmanuel Richard, Isabelle Redonnet-Vernhet, Samir Mesli, Louis Lebreton

**Affiliations:** ^1^ Hôpital Pédiatrique, Pôle Pédiatrique, CHU de Bordeaux, Bordeaux, France; ^2^ Laboratoire de Biochimie, Pôle de Biologie et Pathologie, CHU de Bordeaux, Bordeaux, France; ^3^ lNSERM MRGM U1211, Université de Bordeaux, Bordeaux, France

**Keywords:** inherited metabolic disease, holocarboxylase synthase deficiency, late onset, acidose métabolique, biotin

## Abstract

Holocarboxylase synthase (HCS) deficiency is an extremely rare metabolic disorder typically presenting as severe neonatal metabolic acidosis, lethargy, hypotonia, vomiting, and seizures. This report describes two siblings in a family with late-onset forms of HCS deficiency. The younger sister presented at the age of 11 years and manifested as acute metabolic acidosis, which promptly resolved following rehydration and biotin administration. The results of the organic urine profile confirmed multiple carboxylase deficiency, and genetic testing revealed a novel pathogenic variant in the *HLCS* gene (NM_000411.8) in the homozygous state: c.995A>G; p. (Gln332Arg). No further decompensation was observed for her during the 3-year follow-up period. His older brother was diagnosed at the age of 23 years-old through biochemical tests, without any history of acidotic decompensation. A mini-review of HCS deficiency with late onset (>1 year) or early onset (<1 month) revealed that splice variants are associated with late onset, while both variants p. (Leu216Arg) and p. (Leu237Pro) are associated with early onset. However, the majority of genotypes do not show a clear correlation with the timing of HCS deficiency onset. The most significant point here is the description of extremely late-onset cases of HCS deficiency. This can prompt metabolic investigations and raise suspicion of this rare disease in cases of unexplained metabolic acidosis, even beyond early childhood.

## 1 Introduction

Inherited multiple carboxylase deficiency is a rare autosomal recessive disorder, firstly described in 1971 as biotin-responsive β-methylcrotonylglycinuria (Br med et al., 1945). It was later characterized as a deficiency of four carboxylases: Pyruvate carboxylase (EC 6.4.1.1); Acetyl-CoA carboxylase (EC 6.4.1.2); Propionyl-CoA carboxylase (EC 6.4.1.3) and Methylcrotonyl-CoA carboxylase (EC 6.4.1.4). Biotin is an essential vitamin obtained from diet and from a recycling mechanism. Biotin acts as a coenzyme for the four carboxylation enzymes involved in gluconeogenesis, fatty acid synthesis, and amino acid catabolism.

Two inherited defects have been identified that affect the function of biotin (Sweetman, 1981): Biotinidase and Holocarboxylase synthase deficiency (HCS). Biotidinase deficiency lead to biotin depletion due to an inability to recycle endogenous biotin and to use protein bound biotin from diet (Wolf et al., 1983). In HCS deficiency, biotinylation of the four apocarboxylases is impaired (Burri et al., 1981).

HCS deficiency is a rare metabolic disorder. Newborn screening data allow to estimate an incidence at birth ranging from 1/150 000 (Vilarinho et al., 2010) to 1/300 000 (Niu et al., 2010). The typical presentation is usually a neonatal onset within the first days of life and include lethargy, hypotonia vomiting, seizures and severe metabolic acidosis with hyperlactatemia, ketosis and hyperammonaemia. This metabolic disorder leads to coma and death without biotin supplementation. Patients with less severe defect also present dermatologic signs such as rash, eczema, alopecia and develop psychomotor retardation.

We report the cases of two patients: an 11-year-old girl, referred to as “the first patient,” diagnosed after her first episode of decompensation with typical clinical manifestations, and her older brother, referred to as “the second patient,” diagnosed at 23 years-old. To our knowledge, these represent the latest symptomatic onsets in published cases of HCS deficiency.

## 2 Case description

The first patient is the fourth child of consanguineous parents. She was urgently admitted at 11 years-old to the pediatric emergency department due to lethargy and abdominal pain following 2 days of vomiting. Her past medical history was unremarkable with a normal psychomotor development and no difficulties in school.

On admission, clinical examination revealed a compensated hypovolemia with sinus tachycardia, hypotension, superficial polypnea and cutaneous signs of extracellular dehydration. Consciousness varied, with the Glasgow Coma Scale score ranging between 11 and 15. The patient was somnolent and had no fever. Skin examination was unremarkable and she had no alopecia. Initial investigations revealed a severe metabolic acidosis with ketosis (pH 7.01 and bicarbonate of 3 mmol/L). The biochemical examination showed the following: Hyperlactatemia (8.3 mmol/L), severe hypokalemia (2.18 mmol/L), creatinemia (164 μmol/L) and uremia (12 mmol/L) indicating an acute renal failure. Blood glucose concentration was 1.26 g/L, blood ammonia 28 μmol/L, liver transaminases were normal, with aspartate aminotransferase at 25 U/L and alanine aminotransferase at 15 U/L.

After rehydration and electrolyte correction, the clinical condition improved rapidly and the acidosis resolved almost completely. Lactate decreased to 2.7 mmol/L in few hours.

According to the clinical and biological picture, a presumptive diagnosis of a metabolic inherited disease was suspected.

**FIGURE 1:**
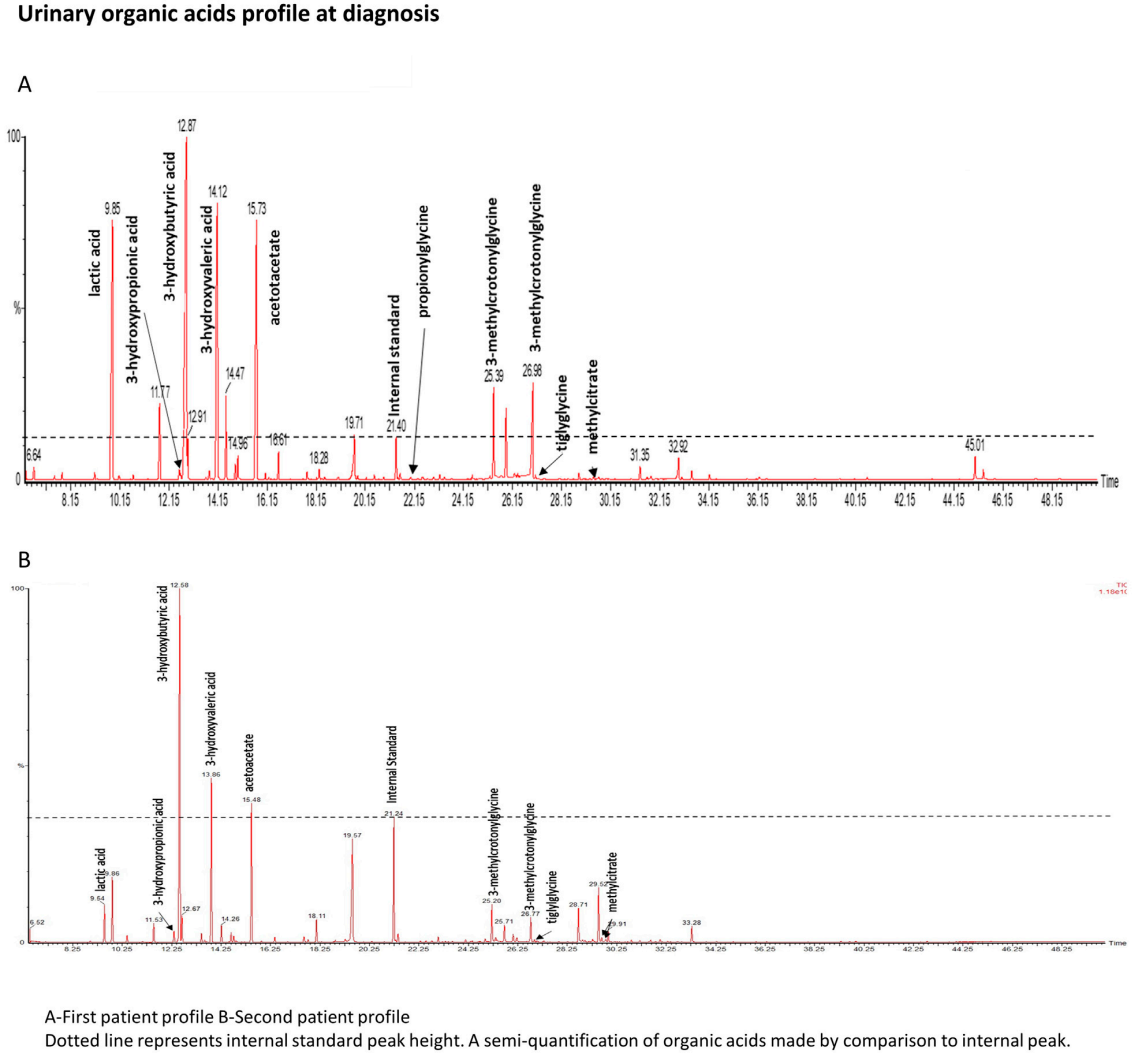
Urinary organic acids profile. Dotted line represents internal standard peak height. A semiquantifiation of organic acids is made by comparison to internal standard peak.

Urinary organic acid analysis was suggestive for profile of an inborn disorder of biotin metabolism with very elevated levels of 3-hydroxyisovaleric acid, 3-hydroxypropionic acid, 3-methylcrotonylglycine and traces of methylcitric acid and tiglylglycine (Figure 1A). These markers were associated with huge peaks of lactate and ketone bodies. Acylcarnitine profile was in agreement with the urinary organic acid profile and showed increased values of 3-hydroxyisovalerylcarnitine (C5OH), propionylcarnitine (C3), tiglylcarnitine and/or 3-methylcrotonylcarnitine (C5:1), acetylcarnitine (C2) and 3-hydroxybutyrylcarnitine (C4OH). The normal serum biotinidase activity (149 nkat/L, within the normal range of 90–210 nkat/L) rules out biotinidase deficiency and suggests a diagnosis of HCS deficiency.

48 h after admission, biotin therapy was started with a dose of 20 mg/day and oral levocarnyl at 100 mg/kg per day.

**FIGURE 2:**
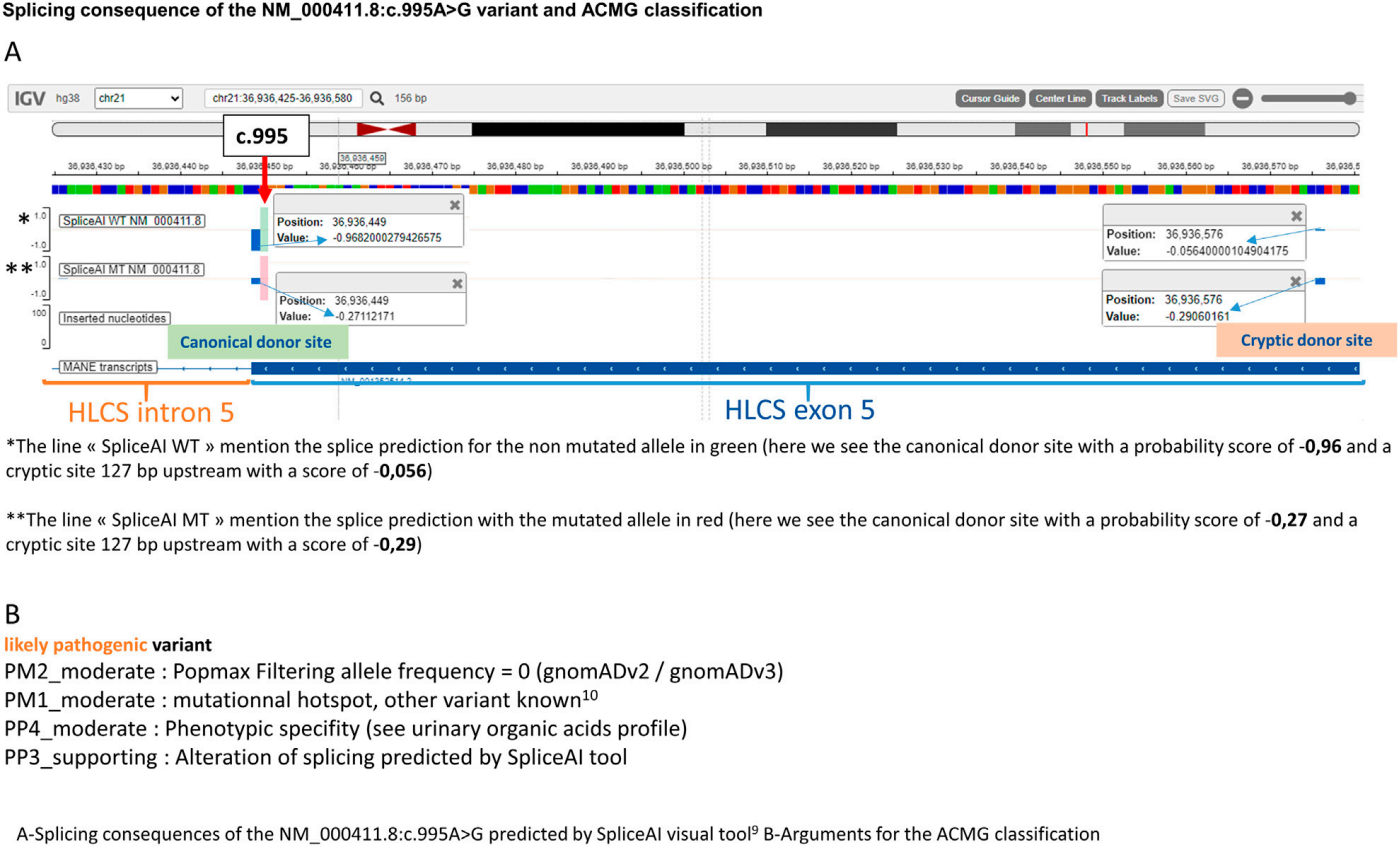
Splicing consequence of the NM_000411.8:c.995A>G variant and ACMG classification.

The diagnosis was later confirmed by genetic analysis which revealed a novel variant on the *HLCS* gene at an homozygous state [NM_000411.8:c.995A>G; p. (Gln332Arg)] that have not been previously reported in the literature. Both parents were tested and found to be carriers of the allele in a heterozygous state. This very rare variant [one heterozygous carrier on gnomAD v2 and one in gnomAD v3 (Chen et al., 2022)] is predicted by spliceAI (Jaganathan et al., 2019; de Sainte Agathe et al., 2023) tool to partially abolish the canonical donor splice site of exon 5 and reinforce a cryptic site located 127 bp upstream (Figure 2A). For the mutated allele, the canonical and cryptic splice sites have a very close probability of being used for the splice, which would lead to one normal mRNA and a shorter one containing a premature termination codon. A previously published variant in the same codon was described in a case of HCS deficiency with a similar predicted effect on the splicing mechanism (Quinonez et al., 2017). Regarding the ACMG classification, the variant was classified “likely pathogenic” (Figure 2B).

The patient was discharged from the hospital after 7 days. After 3 years of metabolic follow-up, metabolic parameters normalized with no further episodes of decompensation, except for a 1-day hospitalization due to an intercurrent infection and vomiting, which resolved rapidly.

The second patient is one of her older brother. His medical history mentions childhood epilepsy, for which he was treated with Valproate until he was 5 years old, a mild global developmental delay, a behavior disorder temporarily treated with neuroleptic and an episodic decrease in visual acuity. No acute episodes were documented. In light of the family history, metabolic investigations were initiated when he was 23 years old, following the diagnosis of his sister. Urinary organic acid (Figure 1B) analysis showed peaks of lactic acid, 3-hydroxybutyric acid and specific markers of a biotin metabolism disorder: 3-hydroxyisovaleric acid, 3-hydroxypropionic acid, 3-methylcrotonylglycine, methylcitric acid and tiglylglycine. These peaks were in smaller proportion than in his sister’s profile confirming that his sample was collecting outside any metabolic decompensation. As his sister, biotinidase activity was normal (186 nkat/L). No acylcarnitine profile or genetic analysis could be performed as the patient was unavailable for these tests. Biotin therapy was started with a dose of 100 mg/day for 1 month then 10 mg/day and oral levocarnyl at 3g/day.

The other two siblings did not exhibit any symptoms and were not tested.

## 3 Materials and methods

### 3.1 Metabolic investigations

Urinary organic profile detection and quantification were performed by gas chromatography–mass spectrometry Clarus 500™ (Perkin Elmer society). After mixing the samples with internal standard solution, organic acids were extracted by liquid/liquid extraction in ethyl acetate. After evaporation, organic acids were derivatized and converted to their corresponding trimethylsilyl ethers by N,O-Bis [trimethylsilyl]trifluoroacetamide.

Flow-injection tandem-mass spectrometry (QUATTRO micro MS/MS, Waters society) with Multiple-reaction monitoring (MRM) mode was used to determine the acylcarnitine profile in dried blood spots. Samples preparation was performed using the Mass Chrom Amino Acids and acylcarnitines 55000 non derivatised Kit (Chromsystems society).

The serum biotinidase activity was performed by a manual colorimetric method that measures conversion of the enzyme’s artificial substrate N-biotinyl p-aminobenzoic acid (B-PABA) (Sigma society).

### 3.2 Molecular analysis of the *HLCS* gene

Genomic DNA was extracted from peripheral blood using an automated method (TECAN Freedom EVO). A panel of 183 genes associated with inborn errors of metabolism, including the HLCS and BTD genes, was applied. The capture encompassed exons and flanking regions (±25 bp) following purification with a SureSelect XTHS2 kit from Agilent Technologies. Sequencing was performed on a Nextseq550 Illumina platform. The bioinformatic workflow employed Alissa Reporter and Alissa Interpret, both from Agilent Technologies. Biological interpretation was carried out according to the ACMG classification.

## 4 Discussion

The cases of two siblings with HCS deficiency have been described, including an eleven-year-old girl who presented with a pattern of decompensation associated with metabolic acidosis, which rapidly resolved with symptomatic management. The late diagnosis of HCS deficiency was confirmed and the patient was managed with oral biotin, without any episodic decompensation 3 years after the diagnosis. The family history revealed no prior instances of metabolic decompensation, such as episodes of metabolic acidosis or related symptoms, before this case. An older brother was then diagnosed through family screening, but he presented an history of non-metabolic clinical symptoms such as epilepsy and mild developmental delay. Interestingly, HCS with isolated epilepsy have already been described (Donti et al., 2016; Suormala et al., 1997).

The variant found was not reported in the literature, but a previously published case from Quinonez et al. (2017) reported neonatal HCS deficiency with a close variant (c.996G>C) abolishing the same splice site at the end of exon 5. However, the clinical impacts of these two cases are difficult to compare since the complete genotype was different: our case is homozygous for the splice variant and the Quinonez et al. (2017) case was in compound heterozygosity for c.996G>C variant and an inversion of chromosome 21 which disrupt the *HLCS* gene.

To investigate an effect of genotype on the age of onset of HCS deficiency, we performed a mini-review of cases at two extremes of age at which the first symptoms of HCS deficiency developed: before 1 month and after 1 year.

The review, conducted in July 2024, identified a total of 20 cases with initial symptoms appearing before 1 month, and 9 cases with symptoms emerging after 1 year. Table 1 summarizes the data from this mini-review.

**TABLE 1:** Mini review of HCS deficiency patients with first symptoms onset before 1 month and after 1 year: genotypes compilations.

First symptoms onset	Genotypes							Number of Subjects	Age(s) at onset	References
	Nature of the genotype	Variant 1			Variant 2					
		Nomenclature^a^	Predictions tools	Functionnal study (Quinonez et al., 2017; Narisawa et al., 1996; Mayende et al., 2012; Dupuis et al., 1999)	Nomenclature^a^	Predictions tools	Functionnal study (Quinonez et al., 2017; Narisawa et al., 1996; Mayende et al., 2012; Dupuis et al., 1999)			
>1 year	SE/SE	c.995A>G; p. (Gln332Arg)	SpliceAI DL = 0,70	none	---------------------------- HOMOZYGOUS ----------------------------			1^b^	11 year	This case report
	SE/SE	c.1519 + 5G>A; p. (?)	SpliceAI DL = 0,38	none	---------------------------- HOMOZYGOUS ----------------------------			1	8 year	Sakamoto et al. (2000)
	MS/MS	c.1522C>T; p. (Arg508Trp)	Revel = 0,897	HLCS Enzymatic activity: moderately decreased (Dupuis et al., 1999)	---------------------------- HOMOZYGOUS ----------------------------			4	19 m, 24 m, 24 m, 20 m	Tang et al. (2003), Hwu et al. (2000), Ling et al. (2023)
	LoF/MS	c.1993C>T; p. (Arg665Ter)			c.500A>C; p. (Tyr167Ser)	Revel = 0,807	Unknown	1	42 m	Donti et al. (2016)
	MS/MS	c.1532A>T; p. (Asn511Ile)	Revel = 0,851	Unknown	c.2078G>C p. (Gly693Ala)	Revel = 0,852	Unknown	1	24 m	Donti et al. (2016)
	MS/MS	c.1648G>A; p. (Val550Met)	Revel = 0,803	HLCS Enzymatic activity: low	---------------------------- HOMOZYGOUS ----------------------------			1	20 m	Aoki et al. (1997)
<1 month	LoF/MS	c.1693C>T; p. (Arg565Ter)			c.977G>A; p. (Gly326Glu)	Revel = 0,959	Unknown	1	18 days	Donti et al. (2016)
	MS/MS	c.647T>G; p. (Leu216Arg)	Revel = 0,856	HLCS Enzymatic activity: null	---------------------------- HOMOZYGOUS ----------------------------			9	1 day	Slavin et al. (2014), Bailey et al. (2008)
	MS/MS	c.710T>C; p. (Leu237Pro)	Revel = 0,956	HLCS Enzymatic activity: null	---------------------------- HOMOZYGOUS ----------------------------			1	1 day	Aoki et al. (1995)
	LoF/MS	c.782del; p. (Gly261ValfsTer20)			c.710T>C; p. (Leu237Pro)	Revel = 0,956	HLCS Enzymatic activity: null	2	1 day, 2 days	Aoki et al. (1995)
	MS/MS	c.721G>T; p. (Gly241Trp)	Revel = 0.963	Unknown	---------------------------- HOMOZYGOUS ----------------------------			2	1 day	De Castro et al. (2015)
	LoF/MS	c.782del; p. (Gly261ValfsTer20)			c.1522C>T; p. (Arg508Trp)	Revel = 0,897	HLCS Enzymatic activity: normal	2	7 days, 19 days	Ling et al. (2023)
	LoF/SE	Paracentric inversion chr21			c.996G>C; p. (Gln332His)	SpliceAI DL = 0,95	PC MCC PCC Enzymatic activity: low	1	1 day	Quinonez et al. (2017)
	LoF/MS	c.782del; p. (Gly261ValfsTer20)			c.1825C > T, p. (Pro609Ser)	Revel = 0.804	Unknown	1	24 day	Ling et al. (2023)
	MS/MS	c.1994G > C; p. (Arg665Pro)	Revel = 0.726	Unknown	---------------------------- HOMOZYGOUS ----------------------------			1	1 m	Ling et al. (2023)

^a^ Nomenclature: HGVS, nomenclature rules for the HLCS, transcript NM_000411.8 (excepted for the chr21 inversion case).

^b^ The older brother was not included because his genotype could not be determined, even though the diagnosis was confirmed through biochemical tests.

Legend: SE, splice effect; MS, missense; LoF = loss of function; SpliceAI DL, donor loss; y = years, m = months, d = days.

Moreover, none of the patients in either group had a genotype with two loss-of-function variants, which probably means that these cases are not viable.

The genotypes between the early and late onset groups are non-concordant, which indicates that the genotypes potentially influence the timing of the condition. The variant p. (Arg508Trp) is an exception, as it was found in both a late onset case (in a homozygous state) and an early onset case (in a compound heterozygous state with a loss-of-function variant).

Some variants are recurrent and are associated either with late cases (variants with partial splicing effect for c.995A>G and c.1519 + 5G>A) or either with early cases [p. (Leu216Arg) and p. (Leu237Pro)]. The other variants appear to be private. Notably, all missense variants excepted one show elevated levels of pathogenic prediction according to the REVEL algorithm (>0,75) (Ioannidis et al., 2016).

It appears feasible to assess phenotype severity through genotype analysis if the aforementioned recurrent variants are detected in the homozygous state. Thus splice variants could be associated with late forms, the p. (Leu216Arg) (Slavin et al., 2014) and p. (Leu237Pro) (Aoki et al., 1995) variants with severe early onset forms. The p. (Arg508Trp) variant is relatively common in late-onset cases, but a publication by Ling et al. (2023) has also demonstrated its frequency in intermediate-onset cases (those occurring between 1 month and 1 year) so we cannot conclude on its severity.

Interestingly, the *HLCS* gene is included in ongoing studies for genetic newborn screening (Holm et al., 2018). This approach can prevent symptoms and complications at early stage, as well as identify late-onset cases at risk of decompensation. A better understanding of the genotype-phenotype correlation would be beneficial, as it would allow for the anticipation of the early or late onset of the condition in the context of genetic newborn screening.

Additionally, prenatal biotin administration may improve the condition in severe early cases if a previous child in the family is known to have an HCS defect (Suormala et al., 1998).

## 5 Conclusion

We presented two cases of holocarboxylase synthase deficiency in a family, both of which were unusually diagnosed at a late stage. The first patient’s outcome was favorable following acute treatment and long-term biotin administration; the second patient did not present specific metabolic signs at 23 years old but a mild developmental delay. Those cases underscore the importance of conducting standard metabolic biochemical assessments when faced with suspicious clinical presentations, even in pre-adolescent children and adults. An analysis of genotypes among cases reported in the scientific literature reveals that the nature of the variants may only partially account for the delayed diagnosis in some patients.

## Data Availability

The datasets for this article are not publicly available due to concerns regarding participant/patient anonymity. Requests to access the datasets should be directed to the corresponding author.
